# Optimization of NIR Spectral Data Management for Quality Control of Grape Bunches during On-Vine Ripening

**DOI:** 10.3390/s110606109

**Published:** 2011-06-07

**Authors:** Virginia González-Caballero, Dolores Pérez-Marín, María-Isabel López, María-Teresa Sánchez

**Affiliations:** 1 Centro de Investigación y Formación Agraria de “Cabra-Priego”, Instituto de Investigación y Formación Agraria y Pesquera (IFAPA), Consejería de Agricultura y Pesca, Junta de Andalucía, Cabra, Spain; E-Mails: virginia.gonzalez.ext@juntadeandalucia.es (V.G.-C.); mariai.lopez.infante@juntadeandalucia.es (M.-I.L.); 2 Department of Animal Production, University of Cordoba, Campus Rabanales, 14071 Cordoba, Spain; 3 Department of Bromatology and Food Technology, University of Cordoba, Campus Rabanales, 14071 Cordoba, Spain

**Keywords:** NIR spectroscopy, quality parameters, on-vine, bunch analysis

## Abstract

NIR spectroscopy was used as a non-destructive technique for the assessment of chemical changes in the main internal quality properties of wine grapes (*Vitis vinifera* L.) during on-vine ripening and at harvest. A total of 363 samples from 25 white and red grape varieties were used to construct quality-prediction models based on reference data and on NIR spectral data obtained using a commercially-available diode-array spectrophotometer (380–1,700 nm). The feasibility of testing bunches of intact grapes was investigated and compared with the more traditional must-based method. Two regression approaches (MPLS and LOCAL algorithms) were tested for the quantification of changes in soluble solid content (SSC), reducing sugar content, pH-value, titratable acidity, tartaric acid, malic acid and potassium content. Cross-validation results indicated that NIRS technology provided excellent precision for sugar-related parameters (r^2^ = 0.94 for SSC and reducing sugar content) and good precision for acidity-related parameters (r^2^ ranging between 0.73 and 0.87) for the bunch-analysis mode assayed using MPLS regression. At validation level, comparison of LOCAL and MPLS algorithms showed that the non-linear strategy improved the predictive capacity of the models for all study parameters, with particularly good results for acidity-related parameters and potassium content.

## 1. Introduction

The achievement of high quality standards in modern wine production depends on harvesting grapes at the optimum stage of ripeness [1]. By determining the right moment for harvesting, winemakers can ensure the best possible wine for any given year or conditions. For this purpose, it is essential to measure a number of grape quality parameters, including soluble solid content, reducing-sugar content, titratable acidity, pH-value, tartaric acid and malic acid contents and sensory attributes [2].

Flanzy [3] has noted that grape organic-acid content determines wine acidity, and also governs the stability, color and acceptability of the final product, since a wine with the right amount of acidity lingers longer on the palate. At the same time, accurate grape-quality measurements enable wineries to stream fruit for crushing and blending, thus maximizing the profitability of their production [4].

Existing analytical methods for the measurement of grape and wine composition do not meet the requirements of modern wine production in a global market, where there is a clear need for fast, accurate, simultaneous and non-destructive measurement of quality parameters both in the raw material and in the finished product [5–7].

Conventional laboratory techniques for measuring different quality characteristics in grapes and wines are tedious, time-consuming and technically demanding, and thus constitute a barrier to the widespread uptake and use of quality descriptors by the grape and wine industry [8].

Because certain variables change in the course of ripening, there is an evident need for non-invasive, objective methods of constantly monitoring the ripening process [9]. These methods can also be used to separate grapes of different qualities at harvest, thus increasing the economic value of the harvest as a whole through product differentiation [10].

The potential of NIRS technology as a non-destructive method for the quantitative characterization of grape quality parameters, using either grape berries or must, has been amply demonstrated [6,10–15]. However, all these studies have required a certain amount of data processing prior to analysis. The authors have thus failed to exploit one of the major advantages of NIRS: the fact that it requires no sample preparation, and is therefore very fast.

Although González-Caballero [7] have addressed the use of NIR spectroscopy models for predicting SSC, reducing sugar content, pH-value, titratable acidity, tartaric acid levels and malic acid content in whole grapes using a spectral range of up to 1,700 nm, the authors stress that the results obtained when analyzing grapes in bunch form should only be considered a first step in the fine-tuning of NIRS technology for on-site control purposes, and that the expectations aroused by NIRS technology for quality control during the ripening process in intact grapes need to be confirmed by increasing the sample set, with a view to improving the specificity, accuracy and robustness of the calibrations obtained.

In practice, however, when new sample groups are included in the calibration set, robustness tends to be increased at the expense of accuracy [16]. One way of overcoming this problem is to develop specific calibrations for small groups of similar samples within the product domain [17]. The method used, known as local regression, combines the advantages of global calibrations obtained using a sample set large enough to ensure coverage of extensive product variation with the accuracy provided by specific calibrations [17].

Dambergs [12] reported improved accuracy in models to predict grape anthocyanin content and pH when sample subsets were selected on the basis of vintage, grape variety and growing region, due to a reduction of calibration non-linearity; in their view, the LOCAL algorithm appears to provide a practical solution to developing robust models for the prediction of these parameters in grapes. They note, however, that the models constructed using MPLS and LOCAL algorithms performed equally well for measuring total soluble solid content.

The aim of this study was to develop accurate and robust NIRS models for measuring major internal quality parameters in intact wine grapes (soluble solid content, reducing sugar content, pH-value, titratable acidity, tartaric acid, malic acid, and potassium content) during ripening and at harvest, regardless of growing season or variety, with a view to enabling growers to routinely use NIRS technology under field conditions to predict more precisely the timing of their harvest operations, and thus ensure the highest possible grape and wine quality.

## 2. Material and Methods

### 2.1. Grape Sampling During Ripening

The sample set for all the parameters tested, except for potassium content, comprised 363 samples of 25 different white and red wine grape varieties (*Vitis vinifera* L.). Grape bunches sourced from experimental vineyards at the Agricultural Research Training Centre at Cabra, near Cordoba (Spain), were harvested in July, August and September in 2006, 2007 and 2008. Grape samples were collected every seven days throughout the study. On arrival at the laboratory, grapes were promptly placed in refrigerated storage at 0 °C and 95% relative humidity. All samples were allowed to stabilize at room temperature (20 °C) prior to Vis–NIR spectral analysis.

### 2.2. Spectrum Collection

Spectra were collected using a Zeiss CORONA portable and non-contact diode-array spectrometer (model CORONA 45VIS/NIR, Carl Zeiss, Inc., Thornwood, NY, USA) equipped with the turnstep module (revolving plate) and a 20-cm-diameter Petri dish to hold the samples, working in reflectance mode in the spectral range 380–1,700 nm, every 2 nm. The measurement distance from the source of light to the sample was 13 mm.

Samples were presented to the instrument in two modes. Spectra were first obtained for intact bunches of grapes. Berries were then passed through a hand-operated food mincer (LI 240, Sammic, SL, Azpeitia, Guipúzcoa, Spain) which enabled constant pressure to be maintained during juice extraction with minimal seed and skin shearing. The must was then centrifuged at 4,000 rpm for 10 min (Centronic 7000577, Selecta, Barcelona, Spain) to remove suspended solids, and the supernatant was used for NIR spectroscopy purposes. A folded-transmission gold reflector cup, diameter 3.75 cm, was used with a pathlength of 0.1 mm.

Eight spectra were captured per sample for each sample presentation mode, and the average of the eight was used in calculations. The signal was captured using CORA software version 3.2.2 (Carl Zeiss, Inc., Thornwood, NY, USA), and subsequently pretreated using the Unscrambler version 9.1 program (CAMO, ASA, Oslo, Norway).

### 2.3. Reference Data Analysis

For each sample, reference data were obtained for SSC, reducing sugar content, pH-value, titratable acidity, tartaric acid, malic acid and potassium content. SSC (°Brix), reducing sugar content, must pH-value, titratable acidity, and tartaric and malic acid contents were measured as indicated by González-Caballero [7]. Potassium content was measured using a CORNING 410 flame photometer (Ciba Corning Diagnostics Limited, Halstead, UK) as previously described by the Spanish Ministry of Agriculture, Fisheries and Food [18]. Results were expressed as milligrams per liter.

### 2.4. Calibration and Validation Sets

The sample set, except for potassium cation, comprised all the available samples (363 samples; 25 varieties) picked during ripening: 108 samples collected in 2006, 120 samples in 2007, and 135 samples in 2008. After eliminating as outliers (n = 2 samples for 2006; n = 4 samples for 2007; n = 13 samples for 2008) those grapes considered over-ripe and thus displaying high sugar content, the initial sample set was divided in two subsets: 251 samples (73% of the total) were used to construct calibration models (calibration set), and the remaining 93 samples (27%), all picked in 2008, were used for external validation (validation set). It should be stressed that the calibration set contained all available samples from 2006 (106 samples) and 2007 (116 samples), together with 29 samples from 2008. These sets were used to develop and subsequently validate models to predict SSC, reducing sugar content, pH-value, titratable acidity, tartaric acid, and malic acid. The calibration and validation sets used to predict potassium content contained only samples from 2008: 104 (80%) for calibration and 44 (20%) for external validation. In all cases, samples were selected solely on the basis of spectral data, following Shenk and Westerhaus [19], using the CENTER algorithm included in the WinISI II software package version 1.50 (Infrasoft International, Port Matilda, PA, USA) prior to developing NIRS calibrations, in order to determine the structure and spectral variability of the study population. This algorithm was applied over the samples belonged to 2008 season.

### 2.5. Chemometric Data Treatment

The WinISI II software package version 1.50 was used for the chemometric treatment of data [20]. Prior to model development using the two regression algorithms (MPLS and LOCAL), different pre-processing combinations were evaluated. As spectral treatments, standard normal variate plus detrending [21] were used to remove multiplicative scatter interferences, and four derivative treatments were tested (1,5,5,1; 2,5,5,1; 1,10,5,1 and 2,10,5,1), where the first number denotes the derivative order, the second denotes the number of nanometers in the segment used to calculate the derivative, and the third and fourth numbers denote the number of data points over which running-average smoothing was conducted [20,22].

First, quantitative calibrations were developed using the MPLS algorithm [23] for predicting internal quality parameters using the bunch as presentation sample; the results were then compared with the calibrations obtained for must. For cross-validation, the calibration set was partitioned into 4 groups; each group was then validated using a calibration developed on the other samples in order to select the optimal number of factors and to avoid overfitting.

The LOCAL algorithm [17] was then used to predict the same quality parameters but using only the bunch as sample presentation, since this is how the winery industry receives the raw material. The LOCAL algorithm operates by searching and selecting from a library (based on the training set) the samples most spectrally similar to the sample to be predicted. The selected samples are used to develop a specific (local) calibration using a modified PLS regression for the prediction of the unknown sample. Selection is based on the coefficient of correlation between the spectrum of the sample to be predicted and each of the sample spectra belonging to the spectral library; those samples displaying the highest correlation are selected.

Different parameters have to be evaluated in order to optimize the LOCAL algorithm [24,25]. In the present study, for each dataset, an optimization design was set up by varying the number of calibration samples (*k*) from 25 to 150 in steps of 25, but including 110, for predicting SSC, reducing sugar content, pH-value, titratable acidity, tartaric acid, malic acid; and from 25 to 75 in steps of 25 for predicting potassium content in bunches; the number of PLS terms (*l*) was varied from 14 to 16 in steps of 1, where the number of predicted values corresponding to the first PLS terms discarded was 4. Finally, the minimum number of samples used for each calibration was set to 15.

For both algorithms, the following spectral regions were tested for calibration purposes: 380–1,650 nm (the highest spectral range with useful information covering the VIS + NIR regions); 780–1,650 nm (the highest spectral range covering the NIR region and including the very near infrared region) and 1,100–1,650 nm (including only the strict near-infrared region). In order to eliminate noise at the end of the spectral range, the region between 1,650–1,700 nm was discarded.

Mahalanobis distance statistics, *H*_global_ and *H*_neighbour_, were computed from the principal components of the selected samples to check the accuracy of predictions [17,26]. The effect of the different settings on the performance of MPLS and LOCAL was evaluated by comparing the standard error of prediction (SEP), the coefficient of regression for the external validation (r^2^), the bias, and the standard error of prediction corrected for bias or SEP(c).

## 3. Results and Discussion

### 3.1. Chemical Composition

During ripening, the study parameters covered a relatively wide range, due to the constant changing of sample matrices. Changes in chemical composition are shown in Table 1, which also indicates the number of samples in the calibration and validation sets following application of the CENTER algorithm, together with mean, standard deviation (SD), and coefficient of variation (CV) values. Samples were collected over the critical months to check for variations in SSC (10.60–58.60 °Brix), titratable acidity (0.20–20.50 g/L tartaric acid) and tartaric acid (4.90–18.60 g/L tartaric acid) in the berry.

The sample set was highly variable, since it contained data from grapes sampled at different stages of ripening over three years; this accounts to a large extent for the high CV values recorded, particularly for SSC, reducing sugar content, titratable acidity, tartaric acid, malic acid and potassium content (Table 1). The results confirm the suitability of this method for selecting the validation set, since calibration and validation set displayed similar values for mean, SD, range and CV for all parameters studied, and the ranges for the validation set lay within the range recorded for the calibration set.

During veraison and until ripening, there is a progressive decline in malic, and to a lesser extent in tartaric acid levels. Flanzy [3] notes that tartaric acid is mostly formed in growing organs and cannot be metabolized except at temperatures over 35 °C. Since temperatures are highest at the end of ripening, it is at this stage when the lowest tartaric acid levels are recorded. Malic acid is synthesized following the combustion of sugars in chlorophyll-containing tissues. Unlike tartaric acid, it is unstable, and is metabolized during ripening, leading to low levels at harvest.

Potassium is the major mineral cation in grapes and plays a major role in the neutralization of tartaric and malic acid in the berries, thereby affecting the grape’s acid profile [27,28]. Potassium directly determines the pH not only of wine but also of must [29]. It is present in wines mainly as potassium bitartrate, an unstable compound that can precipitate at cool temperatures as a crystalline deposit [30].

### 3.2. Calibration Development using MPLS Regression and NIR Spectra

#### 3.2.1. Prediction of Sugar-Related Quality Parameters in Grapes

Table 2 shows the best calibration models obtained using the global set (n = 251) for the prediction of SSC and reducing sugar content according to the spectral range and derivative treatment used, for bunches and musts, using the MPLS algorithm.

The equation displaying the greatest predictive capacity for SSC in bunches was that obtained over the broadest spectral range, *i.e.*, 380–1,650 nm, with statistical values of r^2^ = 0.94; SECV = 1.00 °Brix; RPD = 4.12; CV = 5.08%. The predictive capacity of this equation was slightly poorer than that of the equation obtained with grape must (RPD = 4.29; CV = 4.81%). However, both models displayed excellent predictive capacity in term of the criteria outlined by Williams [31], who suggest than an r^2^ value greater than 0.9 and RPD values greater than 3 indicate excellent quantitative information.

For reducing sugar content, the equation displaying the greatest predictive capacity in bunches was obtained over the range 780–1,650 mm, yielding statistical values for r^2^, SECV and RPD slightly higher than those obtained for must samples. The RPD value (3.95) together with the r^2^ value (0.94) for bunches demonstrated the robustness and power of the calibration models.

No previously-published studies address the direct measurement of SSC in bunches, and values reported for measurements in berries tend to be lower than those obtained here; Larraín [13], for example, recorded an RPD value of 3.40. Kemps [10] reported a RPD value of 5.05 when measuring sugar concentrations in grape berries.

The literature contains only one report evaluating the use of NIRS to measure internal sugar-related quality parameters in grape bunches [7]. The results obtained for SSC (r^2^ = 0.89; SECV = 1.41 °Brix; RPD = 2.92) and reducing sugar content (r^2^ = 0.87; SECV = 17.13 g/L; RPD = 2.77) were lower than those recorded here, confirming the need to work with broad-based sample sets which reflect existing variability, with a view to increasing model robustness and precision. Williams [31] and Pérez-Marín [32] highlight the importance of both sample set size and sample distribution within the calibration set, noting that sample sets for calibration should ideally ensure uniform distribution of composition across the range of the study parameter in question.

#### 3.2.2. Prediction of Acidity-Related Quality Parameters in Grapes

For pH-value, the best statistics (r^2^ = 0.87; SECV = 0.12; RPD = 2.73) for bunch analysis were obtained with the first derivative treatment in the spectral range 380–1,650 nm (Table 2). The value obtained for r^2^ (0.87) would, according to the guidelines put forward by Williams [31], provide sufficiently good quantitative information to enable the classification of musts obtained from these grapes, thus allowing musts to be adjusted prior to fermentation. Interestingly, bunch analysis yielded better results than must presentation for pH-value: RPD = 2.73 and CV = 3.60% for bunch mode and RPD = 1.58 and CV = 6.39% for must presentation.

The results obtained using bunch analysis were better than those obtained by González-Caballero [7] (r^2^ = 0.69; SECV = 0.19; RPD = 1.81), by Cozzolino [33] (RPD = 1.4) and by Larraín [13] (RPD = 2.2); in both these studies, samples were presented in berry form.

Models constructed to predict other acidity-related parameters in bunches (Table 2) may be considered good, as indicated by the values obtained for the determination coefficient (r^2^ = 0.83 for titratable acidity; r^2^ = 0.78 for tartaric acid; and r^2^ = 0.73 for malic acid) [31]. It should also be stressed that the best equations for titratable acidity (r^2^ = 0.83; SECV = 1.07 g/L; RPD = 2.40) and tartaric acid content (r^2^ = 0.78; SECV = 1.18 g/L; RPD = 2.11) were obtained using bunch rather than must analysis. Although better results were obtained for malic acid content using must analysis (r^2^ = 0.78; SECV = 0.74 g/L; RPD = 2.13), the statistics obtained for bunch analysis indicated a fairly similar predictive capacity (r^2^ = 0.73; SECV = 0.81 g/L; RPD = 1.94).

As was the case with sugar-related parameters, and due to an increase in the number and variability of the samples in the calibration set, the models constructed here displayed greater predictive capacity than those obtained by González-Caballero [7] who, in a study of bunch presentation using a set of 108 samples, reported the following values: titratable acidity RPD = 1.35, CV = 24.63%; tartaric acid content RPD = 1.38, CV = 16.02%; and malic acid content RPD = 1.28, CV = 88.42%.

#### 3.2.3. Prediction of Potassium Content in Grapes

The predictive capacity of the models constructed to predict potassium content both in bunches and in must was relatively poor. For bunch analysis, statistical values (r^2^ = 0.44; SECV = 242.26 mg/L) indicated that models were sufficient to distinguish between samples containing high and low levels of potassium [31]. It should be noted that the calibration set available comprised only samples picked in 2008; it is therefore reasonable to assume that—as was the case with sugar-related and acidity-related parameters—the predictive ability of the models could be improved by increasing the number of samples and the variability of sample sets by using samples picked in successive years.

Potassium is the major mineral cation in grapes. Potassium levels thus influence the grape’s acid profile and thus the final quality of the wine obtained. Simultaneous measurement of potassium levels along with other internal quality parameters is therefore clearly of interest to the wine industry.

There are no previous reports on the use of NIR spectroscopy to measure potassium content in grapes, even though information on crop nutrition is essential for winegrowers, enabling them to adequately establish nutrient requirements and to fine-tune fertilizer rates. Sauvage [34], however, have measured potassium levels in wine using this method (r^2^ = 0.86; SECV = 79.00 mg/L).

### 3.3. Comparison of Grape Internal Quality Parameters using MPLS versus LOCAL Algorithms

**Figure 1.**
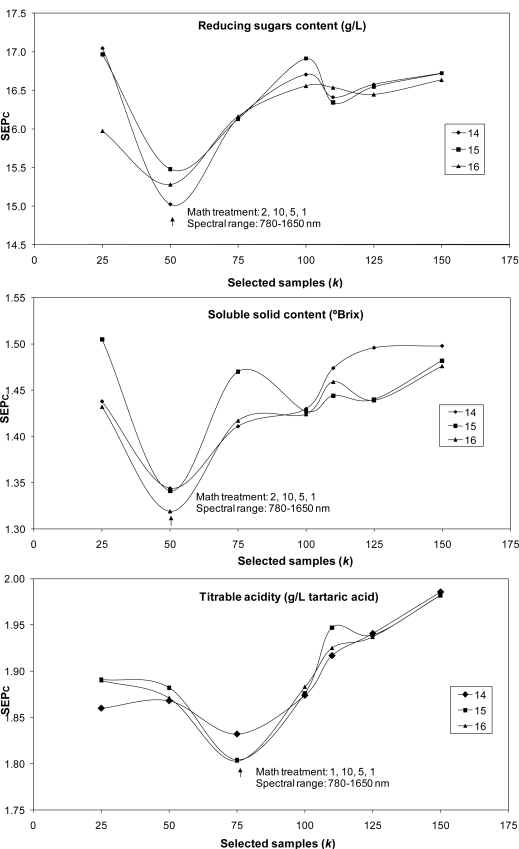

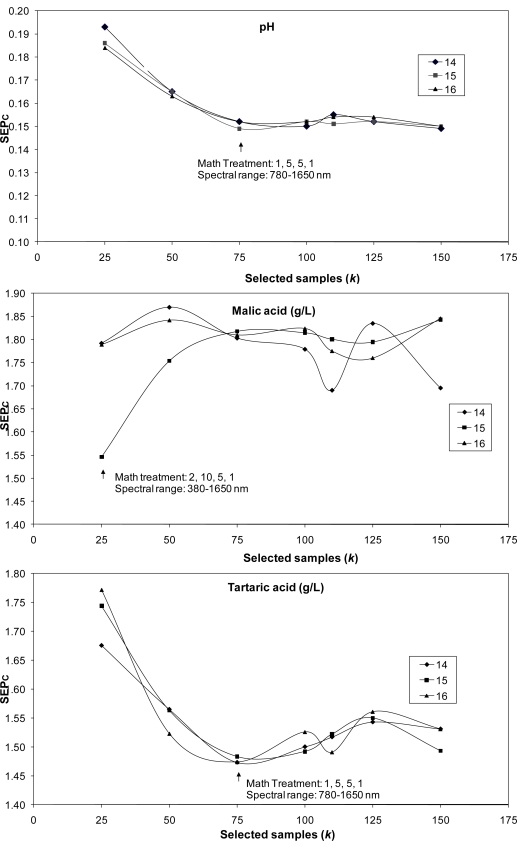

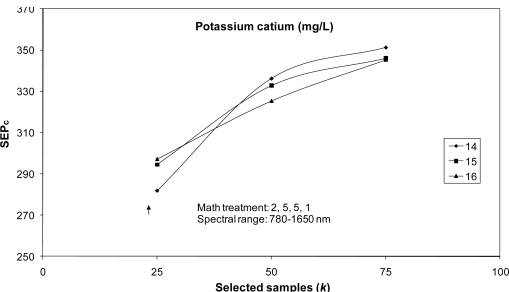
Best SEP_c_ values for the prediction of quality parameters in intact grapes using the LOCAL algorithm for the different selected samples values (*k*), PLS factors (*l*), the best mathematic treatments developed and spectral ranges. ( 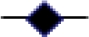) 14 PLS factors; ( 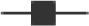) 15 PLS factors, ( 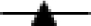) 16 PLS factors).

The LOCAL algorithm was also used to predict internal quality parameters in bunches. The best SEPc values obtained with the best mathematical treatments and spectral ranges over 21 runs (7 values for *k* and 3 for *l*) are shown in Figure 1. The lowest values for SEPc were achieved with the lowest values for *k*. For application of the LOCAL strategy, only 25 samples were used to predict malic acid and potassium content; 50 samples were used for SSC and reducing sugar content predictions, and 75 samples were used for pH, titratable acidity and tartaric acid. Samples were selected as being the most representative of the calibration set, rather than using all the 251 samples or the 104 samples in the case of potassium cation employed to construct the calibration model using MPLS regression.

In most cases, the values selected for parameter *l* (number of PLS terms) had little influence on SEPc values; marked differences being observed only for SSC and malic acid. The best results achieved with the LOCAL algorithm were then compared with those obtained using MPLS regression for the prediction of the 93-sample validation set except for the potassium cation (n = 44), using only bunch analysis (Table 3).

It needs to point out that the sistematic procedure follows in this work is more favourable for the LOCAL algorithm, since the tuning of LOCAL was done on the validation set, with 21 combinations of parameters, being selected the one with best SEP, while the best MPLS model was chosen taking into account the cross validation statistics and only the best model applied to the validation set. Nevertheless, this systematic has been selected since the differences between the statistics obtained for the MPLS models were no-relevant, and also taking into account that cross-validation statistics, according to Shenk [23], give a realistic estimate of the error prediction of samples not included in the calibration.

The results obtained using LOCAL were better than those achieved using MPLS regression, in terms of both r^2^ and SEPc, for total soluble solid content, reducing sugar content, pH, tartaric acid and potassium content, although the improvement was only slight for reducing sugar and tartaric acid content. For titratable acid and malic acid, application of the LOCAL algorithm improved model precision but also prompted a slight increase in SEPc values.

The improvement in r^2^ values achieved using the non-linear strategy ranged between 2% for both tartaric acid and reducing sugar content and 41% for malic acid, while the improvement in SEPc values ranged from 1.5% for tartaric acid to 22% for SSC. Shenk [35] suggest that application of the LOCAL algorithm can improve the predictive capacity of models by 10–30%.

With regard to spectral range suitability, the best results using the LOCAL strategy were generally obtained in the 780–1,650 nm range, except for malic acid content; by contrast, MPLS yielded the best results for all parameters using a wider spectral range (380–1,650 nm), except for reducing sugar content. The optimal number of PLS terms for the prediction of each parameter using the LOCAL algorithm (Table 3) was always equal to or smaller than the optimal number using MPLS.

## 4. Conclusions

The results obtained here when analyzing grapes in bunch form—a method that requires no previous sample preparation—confirm that NIRS technology is well suited for evaluating internal quality characteristics related to sugar content and acidity, for the non-destructive quantification of chemical changes taking place during on-vine ripening, and for deciding on the optimum time for harvesting. NIR technology additionally enables the classification of bunches in terms of low *versus* and high potassium levels, using a very fast, non-destructive sensor.

The results also highlight the need to develop models using a database sufficiently large to reflect the spectral variability that may be encountered during on-vine ripening. In comparison with MPLS regression, the LOCAL algorithm proved to be a highly effective tool for improving the prediction of internal quality parameters in intact grapes.

## Figures and Tables

**Table 1.** Statistical analysis of calibration and validation sets: data range, mean and standard deviation (SD) and coefficient of variation (CV).

**Parameter**	**Item**	**Calibration Set (n = 251 Except Potassium, n = 104)**	**Validation Set (n = 93, Except Potassium, n = 44)**
Soluble solid content (°Brix)	Range	10.60–58.60	11.80–27.50
	Mean	20.49	19.88
	SD	5.84	3.77
	CV (%)	28.51	18.96
Reducing sugar content (g/L)	Range	81.50–586.40	114.30–287.00
	Mean	198.39	203.88
	SD	64.95	41.58
	CV (%)	32.74	20.40
pH-value	Range	2.48–4.60	2.60–3.80
	Mean	3.35	3.33
	SD	0.34	0.25
	CV (%)	10.19	7.60
Titratable acidity (g/L tartaric acid)	Range	0.20–20.50	3.40–19.10
	Mean	6.72	6.42
	SD	3.52	3.07
	CV (%)	52.29	47.79
Tartaric acid (g/L tartaric acid)	Range	4.90–18.60	7.30–15.70
	Mean	9.48	9.74
	SD	2.80	1.96
	CV (%)	29.53	20.14
Malic acid (g/L malic acid)	Range	0.10–14.50	0.30–13.10
	Mean	2.33	2.74
	SD	2.32	2.51
	CV (%)	99.71	91.85
K (mg/L)	Range	841.00–2,737.00	938.00–2,522.00
	Mean	1,692.28	1,675.99
	SD	401.12	340.02
	CV (%)	23.70	20.29

**Table 2.** Calibration statistics for the models obtained for predicting soluble solid content (SSC), reducing sugar content, pH-value, titratable acidity, tartaric acid, malic acid and potassium content for the different sample presentations and spectral ranges studied (calibration set, n = 251 samples except for potassium cation, n = 104) using MPLS regression.

**Parameter**	**Sample Presentation**	**Spectral Range (nm)**	**Mathematic Treatment**	**Mean^1^**	**SD^2^**	**SEC^3^**	**R^2^^4^**	**SECV^5^**	**r^2^^6^**	**RPD^7^**	**CV(%)^8^**
SSC (°Brix)	Bunch	380–1,650	2,5,5,1	19.71	4.13	0.74	0.97	1.00	0.94	4.12	5.08
	Must	780–1,650	1,10,5,1	19.92	4.11	0.86	0.96	0.96	0.95	4.29	4.81*
Reducing sugar content (g/L)	Bunch	780–1,650	2,5,5,1	191.72	53.80	10.76	0.96	13.63	0.94	3.95	7.11*
	Must	380–1,650	2,5,5,1	195.48	51.14	12.00	0.94	15.36	0.91	3.33	7.86
pH-value	Bunch	380–1,650	1,10,5,1	3.34	0.33	0.10	0.91	0.12	0.87	2.73	3.60*
	Must	380–1,650	1,5,5,1	3.36	0.34	0.20	0.65	0.21	0.60	1.58	6.39
Titratable acidity (g/L tartaric acid)	Bunch	380–1,650	1,10,5,1	6.11	2.57	0.96	0.86	1.07	0.83	2.40	17.49*
	Must	380–1,650	1,5,5,1	5.94	2.48	0.82	0.89	1.11	0.80	2.24	18.62
Tartaric acid (g/L tartaric acid)	Bunch	380–1,650	1,10,5,1	9.20	2.49	1.08	0.81	1.18	0.78	2.11	12.78*
	Must	380–1,650	1,5,5,1	8.90	2.36	1.21	0.74	1.28	0.71	1.85	14.35
Malic acid (g/L malic acid)	Bunch	380–1,650	2,5,5,1	1.86	1.57	0.68	0.82	0.81	0.73	1.94	43.48
	Must	380–1,650	2,10,5,1	1.85	1.57	0.56	0.87	0.74	0.78	2.13	39.69*
K (mg/L)	Bunch	380–1,650	1,5,5,1	1,634.35	324.74	193.06	0.65	242.26	0.44	1.34	14.82*
	Must	380–1,650	1,5,5,1	1,676.75	319.49	242.97	0.42	258.94	0.35	1.23	15.44

1 mean of the calibration set;

2 standard deviation;

3 standard error of calibration;

4 coefficient of determination of calibration;

5 standard error of cross validation;

6 r^2^: coefficient of determination of cross validation;

7 ratio SD/SECV;

8 coefficient of variation;

* best equation.

**Table 3.** Validation statistics for the best models for predicting soluble solid content (SSC), reducing sugar content, pH-value, titratable acidity, tartaric acid, malic acid and potassium content using MPLS and LOCAL algorithms.

**Parameter**	**Method**	**Mathematic Treatment**	**Spectral Region**	**Factors**	**SEP^1^**	**SEPc^2^**	**Bias**	**r^2^^3^**	**Slope**
SSC (°Brix)	MPLS	2,5,5,1	380–1,650	16	1.69	1.69	0.17	0.80	0.97
	LOCAL (*k* = 50)	2,10,5,1	780–1,650	16 (−4)	1.33	1.32	0.24	0.88	0.96
Reducing sugar content (g/L)	MPLS	2,5,5,1	780–1,650	16	16.67	15.80	5.57	0.86	0.94
	LOCAL (*k* = 50)	2,10,5,1	780–1,650	14 (−4)	16.40	15.02	6.77	0.88	0.91
pH-value	MPLS	1,10,5,1	380–1,650	16	0.17	0.17	0.02	0.58	0.84
	LOCAL (*k* = 75)	1,5,5,1	780–1,650	15 (−4)	0.15	0.15	0.02	0.66	1.11
Titratable acidity (g/L tartaric acid)	MPLS	1,10,5,1	380–1,650	16	1.73	1.67	−0.49	0.48	0.85
	LOCAL (*k* = 75)	1,10,5,1	780–1,650	16 (−4)	1.87	1.80	−0.54	0.66	0.97
Tartaric acid (g/L tartaric acid)	MPLS	1,10,5,1	380–1,650	16	1.60	1.49	0.60	0.46	0.88
	LOCAL (*k* = 75)	1,5,5,1	780–1,650	14 (−4)	1.47	1.47	0.08	0.47	0.79
Malic acid (g/L malic acid)	MPLS	2,5,5,1	380–1,650	16	1.39	1.39	0.20	0.30	0.95
	LOCAL (*k* = 25)	2,10,5,1	380–1,650	15 (−4)	1.54	1.55	−0.02	0.51	0.89
K (mg/L)	MPLS	1,5,5,1	380–1,650	14	300.23	301.15	−38.83	0.29	0.67
	LOCAL (*k* = 25)	2,5,5,1	780–1,650	14 (−4)	284.52	281.71	−69.02	0.39	0.80

1 standard error of prediction;

2 standard error of prediction bias-corrected;

3 coefficient of determination of prediction.
